# Exploring the perceived impact and influence of women leaders across sub-Saharan Africa on health policy and gender equity

**DOI:** 10.1136/bmjgh-2025-021163

**Published:** 2025-12-19

**Authors:** Anna Kalbarczyk, Milly Nakatabira, Katherine Banchoff, Islam Ahmed, Sualiha Abdulkader, Terefe Gelibo Argefa, Choolwe Jacobs, Helen Kuo, Malanto Rabary, Rosemary Morgan

**Affiliations:** 1 International Health, Johns Hopkins University Bloomberg School of Public Health, Baltimore, Maryland, USA; 2 International Institute for Primary Health Care, Addis Ababa, Ethiopia; 3 Epidemiology and Biostatistics, University of Zambia, Lusaka, Zambia; 4 Independent Consultant, Antananarivo, Madagascar

**Keywords:** global health, health systems, immunisation, public health, decision making

## Abstract

**Introduction:**

Women make up a significant portion of the global health workforce but are under-represented in leadership roles. In sub-Saharan Africa (SSA), 70% of the health workforce is women, yet only 38% hold leadership positions in health ministries. This gap can lead to gender biases in health research and policymaking, perpetuating systemic gender biases. Despite these barriers, women leaders are making an impact globally. However, evidence of their impact is lacking.

**Methods:**

We conducted an explanatory sequential mixed-methods study to identify perceptions of women leaders’ influence and impact within the fields of reproductive, maternal, child and adolescent health and nutrition and immunisation across SSA. The study included a multicountry online survey with men and women leaders and key informant interviews with a subset of women leaders. Descriptive statistics were computed with SPSS, and thematic analysis was conducted using NVivo.

**Results:**

408 women and men leaders completed at least one section of the survey; 34 women leaders participated in key informant interviews. Women leaders are conducting their leadership differently, using identity-derived power and the power of the collective to influence health policy and programme change. They have unique access to communities and can build trust with marginalised groups. Women leaders also prioritise women-centric and neglected health issues, demonstrating ethical responsibility through transparency, commitment to inclusion, accountability and maximising impact with limited resources.

**Conclusion:**

The findings from this study underscore the critical role of women leaders in advancing health policy and gender equity across SSA. Women leaders’ impact can be amplified and enhanced through targeted investments that strengthen enabling environments, foster allyship, champion gender integration activities implemented by women leaders and support their unique networks. Such investments will benefit women and adolescent girls and contribute to achieving broader public health goals and sustainable development.

**WHAT IS ALREADY KNOWN ON THIS TOPIC**

- Despite being a significant part of the health workforce, women are under-represented in leadership roles, leading to gender biases in research and policymaking.
- Despite the many well-documented barriers they face—or perhaps in response to them—women leaders are making an impact.

**WHAT THIS STUDY ADDS**

- This is the first study of its kind to generate empirical evidence on the impact of women leaders.
- Women leaders are using different approaches and strategies to positively influence health policy and programme changes for women, children and vulnerable populations.

**HOW THIS MIGHT AFFECT RESEARCH, PRACTICE OR POLICY**

- Investments in women’s leadership can have an important impact on the development, implementation and prioritisation of programmes and policies that seek to target women, children and vulnerable populations.

## Introduction

 Investing in women’s leadership in global health is crucial for achieving equitable health outcomes and advancing sustainable development goals. Women constitute a significant portion of the global health workforce, yet they remain under-represented in leadership roles.^1 2^ In sub-Saharan Africa (SSA), about 70% of the health workforce is made up of women, yet only 38% of them hold leadership positions in health ministries.^3^ This gap can lead to gender biases in health research and policymaking, perpetuating systemic gender biases that overlook the unique needs of women and marginalised populations.^4 5^ Women leaders in SSA are navigating high-level decision-making spaces that are mostly dominated by men, such as boards, professional societies, parliament and national assemblies. They face unique challenges in their roles as they navigate these spaces, which are deeply rooted in household, institutional and societal norms and cultures that perpetuate gender inequality.^6^ Such barriers include but are not limited to gender bias and discrimination, sexual harassment, lack of representation, women’s second shift as care providers to their families, limited access to networks and mentorship and funding and resource inequities.^2 4 7^

Despite these barriers, or in response to them, we know that women leaders are making an impact and facilitating improved health and wellness for people globally. Women leaders in global health implement policies that are more supportive of women and children,^8^ and their representation in local politics can foster enhanced career aspirations among parents of girls.^9^ A recent literature review including 137 articles found that women leaders across geographies and organisation types have positive impacts on financial performance, innovation, engagement with ethical and sustainability initiatives, health, organisational culture and climate and influence on other women’s careers and aspirations.^10^ Even those studies reporting mixed findings still largely pointed to positive results, particularly when modified by other factors, such as better education, greater levels of experience and opportunities to work with other women across an organisation. However, evidence on women leaders’ impact on gender inequity, health policy and health outcomes is lacking.

The Global Financing Facility (GFF) was launched in 2015 to help address gaps in financing for health systems and interventions needed to improve Reproductive, Maternal, Newborn, Child and Adolescent Health and Nutrition (RMNCAH-N) outcomes. The GFF strategy promotes an *increased focus on gender equality and country leadership*. To inform investments in national and regional leadership, the GFF and Gavi, the Vaccine Alliance are collaborating to support evidence generation on the role and impact of women’s leadership in the health sector. This manuscript reports results from Transforming Health: The role and impact of women’s leadership in the health sector (THRIVE) project. THRIVE sought to document the depth and breadth of women leaders’ impact across SSA on prioritisation of key issues related to women’s health policy and programmes, including RMNCAH-N and immunisation.

## Methods

Between June and September 2024, we conducted an explanatory sequential mixed-methods study including an online survey with men and women leaders, followed by key informant interviews (KIIs) with a subset of women survey participants. We used the Good Reporting of A Mixed Methods Study checklist to prepare this manuscript.^11^

### Survey

Participants were included in the survey if they self-identified as a leader in RMNCAH-N and/or immunisation. We defined leadership as “those who occupy a position which gives them *influence and power over identifying priorities, providing strategic direction, allocating resources and decision-making* within the immunisation and/or RMNCAH-N sector at either the sub-regional, regional, national, or continental level”. To identify as a leader, participants needed to answer ‘yes’ to one or more of the following questions—In your current role, do you have influence over: (1) how decisions are made, (2) which priorities are identified, (3) how funding is distributed or (4) the strategic direction of your organisation or institution?

The tool was developed collaboratively with input from all study team members and is included in the online supplemental material. Results from a recently conducted literature review identified key areas of women leaders’ impact across a range of sectors, including health^10^; these impact areas informed the items in the survey. A draft tool was piloted with four women leaders with expertise in immunisation and RMNCAH-N. Their feedback was used to clarify items and reduce the length of the tool wherever possible.

The survey was designed in Qualtrics, an online survey programme, and distributed via email to leaders identified during a comprehensive stakeholder mapping exercise. We developed a stakeholder mapping methodology (described elsewhere) to ensure accurate identification and representation of leaders in RMNCAH-N and immunisation in SSA who could serve as a sampling universe.^12^ Leaders were recruited via email and LinkedIn using a recruitment script.

Participants provided informed consent following a description of the study purpose and objectives. They were then asked to identify the extent to which they agreed with a series of statements about women leaders’ impact on key organisational, programme, policy, gender and health outcomes.

Women and men leaders were recruited across SSA. To ensure adequate representation, we calculated a sample size of 625 respondents. The sample size was determined based on sample sizes used in similar research and by applying the sample size estimation formula for frequency in a population with correction for smaller population size (sample size n=[DEFF×Np(1−p)]/[(d2/Z21−α/2×(N−1)+p×(1−p)]), with sample size assumptions being 95% level of confidence and 80% power, with a design effect of 1.5. The limits of the sample size calculation also considered the accessibility and willingness of the participants to take the women’s leadership survey. Similarly, lengthy internet surveys have shown dropout rates between 2% and 10%, which were considered when calculating sample size.^13^

While we sought to ensure that 50% of the sample size were women leaders, we recognised that on a global scale, due to the lack of representation of women leaders, we were more likely to capture 25% and aimed for a minimum of 25% of women respondents. This 25% is in line with the current proportion of approximately 25% of women in leadership roles in global health. The survey’s sample size was determined by those who met the eligibility criteria and completed at least one survey section. While 742 individuals opened the survey and met eligibility criteria, the final sample size was 408, which included respondents who met the eligibility criteria and answered at least one survey section. Because response rates varied across sections and individual items, the total number of valid cases is reported for each section as appropriate. Despite the lower-than-expected overall response rate, the final sample remains statistically adequate, as recommendations for minimum sample sizes in health and implementation research suggest that having at least 100 participants per analytic group is sufficient for reliable estimation and robust analysis.^14^

Since this was an online survey, we anticipated a substantial proportion of invitees might not respond. To address this, we invited a considerably larger number of participants than the target number of surveys completed by our sample size calculation. In our stakeholder mapping, we identified and reached out to 3901 potential participants, some of whom forwarded emails to others in their networks. On average, 81 stakeholders were identified for each country. Approximately 38% (n=1353) of the identified individual stakeholders were women. This over-recruitment was designed to ensure that, even with non-response, we would achieve the desired sample size for adequate statistical power and representativeness.

### Survey analysis

The data were exported into the SPSS for Windows for analysis. Although 408 total respondents were eligible and completed at least one survey section, the survey was designed for respondents to skip questions if they chose; therefore, the descriptive statistics, including means, SD, frequencies and percentages, were calculated per question based on the number of respondents who answered the survey question. This is due to the large variability in the number of respondents who answered each survey question, and results are reported based on question responses rather than survey responses. For categorical or nominal data, frequencies and percentages were reported, where frequency represents the number of participants in a specific category, and percentage indicates the proportion of the sample in that category. For interval or ratio data, means and SD were computed. Participants rated their beliefs about the impacts of women leaders using a Likert scale ranging from 1 (strongly disagree) to 5 (strongly agree). Beliefs regarding women leaders’ impact were assessed across three domains. Organisational outcome characteristics were measured using nine items capturing perceptions of women leaders’ influence within organisations. Overall outcome characteristics were measured using nine items assessing perceived impact on general outcomes. Women’s health and gender outcomes were measured using 12 items evaluating beliefs on women leaders’ influence on health-related and gender-related outcomes. All items used a 5-point Likert scale (1=strongly disagree, 5=strongly agree), with higher total scores indicating stronger perceived positive impact. Item responses were summed within each domain to generate total scores, allowing meaningful comparisons across respondents and subgroups. These outcome domains were selected because they reflect key areas through which women leaders may influence RMNCH-N and immunisation programme performance. Analyses were conducted per question using variable denominators to account for item non-response, and the number of valid responses per item is reported to ensure transparency regarding precision and potential bias. To explore participant agreement further, responses of ‘strongly agree’ and ‘agree’ were combined to indicate agreement, while ‘strongly disagree’ and ‘disagree’ were merged to indicate disagreement. Data were analysed per question using variable denominators to account for item non-response across sections. This approach ensured the inclusion of all available data for each variable, maximising the use of valid responses while minimising unnecessary case exclusion. However, varying response rates across items may have influenced the precision of some estimates.

Descriptive findings were disaggregated by gender, primary affiliation and African regions. The independent t-test was conducted for comparisons of the mean scores for two groups (women and men), whereas one-way analysis of variance (ANOVA) was conducted to compare the difference in means between two or more groups using the F-distribution. Prior to conducting these tests, assumptions of normality and homogeneity of variance were evaluated. Normality was assessed using Shapiro-Wilk tests and by examining skewness and kurtosis values. Homogeneity of variance was tested using Levene’s test. In cases where assumptions were marginally violated, analyses were repeated using Welch’s ANOVA and non-parametric equivalents to confirm robustness. Statistical significance was set at p<0.05 for all tests.

### Key informant interviews

KIIs were conducted among women leaders who responded to the survey and indicated interest and willingness to participate in a follow-up conversation. Interviews were conducted based on availability and oral consent. Interviews were conducted in English and lasted approximately 45–60 min. Interview guides focused on how women leaders have contributed to and/or influenced agenda-setting and priorities, funding and how it is distributed, related health outcomes and policies. These were all outcome categories explored in the survey. All interviews were audio recorded and transcribed.

Saturation was assessed collaboratively by the research team during debriefing sessions and ongoing analysis. The team monitored emerging themes through iterative coding, and saturation was confirmed when no new significant themes or insights appeared across consecutive interviews or data segments. Decisions regarding saturation were made through consensus discussions, where team members reviewed coding completeness and the stabilisation of themes. Data collection concluded once the team agreed that additional data were unlikely to contribute new information, indicating that thematic saturation had been reached.

### KII analysis

A thematic analysis of all qualitative data was conducted. The analysis process began with an open coding method, and then a priori themes were defined based on the overall goal of this study, with new themes emerging from the data added to the analysis. The a priori themes for the KII analysis were derived from a previously conducted scoping review on the impact of women leaders.^10^ This review synthesised existing literature on the topic and provided the conceptual foundation for identifying and organising the themes used in our analysis. A codebook was developed through discussions among study team members. The codebook contained both a priori and emergent codes: a priori codes were derived from the study objectives and study guides; emergent codes were identified through reading the transcripts, debriefing and reflections. A cross-coder exercise was conducted using a single transcript to test and refine coders’ understanding of the themes in the codebook. Each transcript was read and coded using NVivo software. Quotes for each code were examined, and matrices and memos were used to organise and examine the information for patterns and to develop emerging interpretations.

We merged findings from the survey and qualitative interviews to generate an impact pathway, highlighting the unique ways in which women use their leadership to influence priorities and affect gender, health and organisational outcomes.

## Results

**Figure 1:**
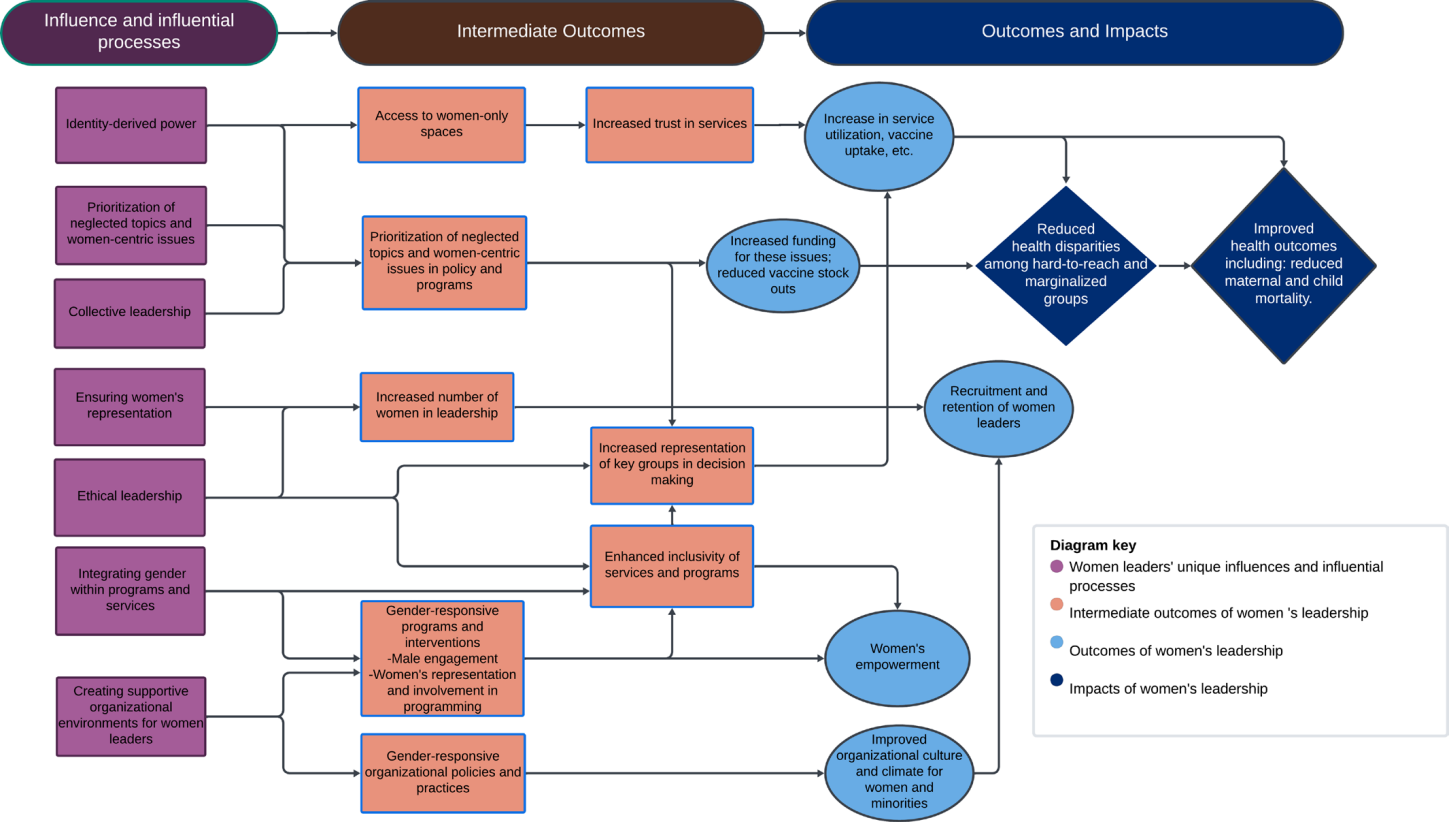
Women leaders’ impact pathway for gender equity and health.

Approximately 408 women and men leaders met eligibility criteria and completed at least one section of the survey. Most survey respondents (58.3%) were women. Organisational representation varied widely, and most participants were currently affiliated with non-governmental organisations (NGOs) (n=190, 46.6%), government (n=106, 26%) or multilateral organisations (n=72, 17.6%). Geographically, most respondents were from Eastern Africa (n=164, 40.2%), followed by Central Africa (n=128, 31.4%), West Africa (n=106, 26%) and Southern Africa (n=10, 2.5%). Respondents from the Democratic Republic of the Congo drove representation from Central Africa (see figure 1).

Thirty-four women leaders were recruited for the KIIs to discuss their areas of impact and influence within the RMNCAH-N and immunisation spaces. One woman who worked extensively in both RMNCAH-N and immunisation was interviewed twice for a total of 35 KIIs. Table 1 displays the demographic data collected for both survey and KII participants.

**Table 1:** Study respondent characteristics

Characteristics	Survey (n=408)		KII (n=35)		Total	
Gender	**N**	**%**	**N**	**%**		
Woman	238	58.3	35	100	273	61.6%
Man	169	41.4	–	–		
Unspecified	1	0.2	–	–		
Organisation type						
Government	106	26.0	10	28.6	116	26.2%
University/Academia	15	3.7	2	5.7	17	3.8%
Non-governmental organisation/Non-profit	190	46.6	15	42.9	205	46.3%
Faith-based organisation	7	1.7	1	2.9	8	1.8%
Private sector	14	3.4	2	5.7	16	3.6%
Multilateral organisation	72	17.6	5	14.3	77	17.4%
Other	4	1.0	0	0	4	0.9%
Geographic region						
Central Africa	128	31.4	2	5.7	130	29.3%
West Africa	106	26.0	7	20	113	25.5%
East Africa	164	40.2	21	60	185	41.8%
South Africa	10	2.5	5	14.3	15	3.4%

KII, key informant interview.

Results focus on women leaders’ unique contributions to RMNCAH-N and immunisation, the outcomes and impacts likely unachievable without them and the influences or processes that help them achieve these results. Figure 1 is a visual depiction of these unique influential processes, related intermediate outcomes and gender equity and health outcomes and impacts.

### Use of identity-derived power

‘Identity-derived power’ refers to the influence or authority a person gains from aspects of their personal identity, such as gender, race, ethnicity or lived experiences. This power is often rooted in the unique perspectives, insights and credibility individuals have because of their identity, which can resonate with or represent specific communities. Identity-derived power allows individuals to shape policies or decisions in ways that reflect their unique vantage points.

Women leaders’ experiences as women, mothers and caregivers often enable them to shape inclusive health policies with a nuanced understanding of the needs of women and children.

[…] women are concerned because we are mothers ourselves; […], some of us have experienced these things ourselves; facility-based delivery. And so, you have an intricate link to the situation; it is not hearsay, and you know how important this is for other women. (WL11_Kenya_R)

Women leaders’ ability to connect with families at a personal level through speaking as a mother had tangible outcomes, including the successful vaccination of over 200 000 children and the management of severe malnutrition cases.

From 2019 to date we have vaccinated many children. We have screened over two hundred thousand children for malnutrition. We have managed more than two thousand severely malnourished children. Since we are mostly employed by the door-to-door, we have time to talk with each family, I realized that talking to them as a mother has really helped them to be able to start changing behaviors. (WL02_Cameroon_IR)

Women’s identity also often provides access to spaces that men traditionally are not allowed to enter (ie, women’s help groups, homes or maternal health centres), allowing them to gain deeper insights into specific issues these groups face, build trust in the community and advance grassroots advocacy.

When we talk about it in a different context, there are some settings that men will not be able to go to, no matter how passionate they are… They cannot reach the woman, and we are talking about our couples-end services here, where we need to reach women. And so, for me, as a female, I do not have the barrier that my male counterparts will have in reaching beneficiaries, which are mostly women and children. (WL06_Nigeria_I)

### Prioritisation of women-centric and/or neglected topics within policy and programming

Women leaders’ engagement often focused on neglected and/or women-centric issues, such as women’s health, or the health of marginalised groups such as persons with disabilities. An overwhelming percentage of question respondents *strongly agreed/agreed (86.4%, n=152/176*) that women leaders positively influence the prioritisation of key issues related to women and girls (84.7%, n=72/85 men and 87.9%, n=80/91 women).

Right now, with other women leaders, we are championing for the government to declare gender-based violence and violence against children a national disaster. We are not succeeding because the men are all over the place saying, ‘Oh, we cannot commit’, but we are not giving up. (W19_Eswatini_I)

This led to the development of policies focused on key women’s health issues, resource mobilisation focused on these issues and programmes and services targeted to women or marginalised groups. For example, one woman leader in Eswatini advocated for the development of sexual and reproductive health strategies and guidelines.

When I joined [my organization], there was no strategy plan for maternal health for these components […]. Like, SRH strategy; there was no strategy, and there were no guidelines, especially the family planning guidelines. There were no obstetric care guidelines. (WL27_Eswatini_R)

We ensure that health budgets at the county level reflect the needs of children and mothers through lobbying efforts. (WL15_Kenya_I)

### Collective leadership

Many women leaders use the power of the collective to overcome male-dominated decision-making spaces. That is, women can leverage their combined leadership power to promote agendas that might face resistance or ambivalence from men. This has contributed to the development of national policy, such as the National Health Act in Nigeria**,** as well as the development of gender-related policies and bills, and the introduction of human papillomavirus vaccines in other countries.

There was a time went for the GEO Bill, the gender and equal opportunity bill. The men refused to follow us at all, at all. They refused to do anything about it, and we gathered women, we carried placards, went there. […] We called the press conference called women, market women. And we tired up and went to the legislator, and they changed it and made it happen. (WL08_Nigeria_IR)

In some cases, women used direct advocacy approaches such as lobbying, protesting, holding press conferences and/or strategically engaging with influential women in their country. These strategies have resulted in increased funding for neglected and women-centric issues.

But collectively, for the country, we are pushing an agenda that everybody should be doing anyway. We feel as the collective, and for us, it is moving the needle. It is moving the needle to include breastfeeding. It is moving the needle to include child nutrition. If you want to talk brain development, you cannot talk brain development without talking about healthy, well-nourished children, and the ECD sector was not talking this language. (WL31_South Africa_R)

### Ethical engagement

Some women leaders show ethical responsibility through transparency, commitment to diversity, equity and inclusion, maintaining accountability and maximising impact even with limited resources. When asked about organisational outcomes, respondents were most likely to strongly agree that women’s leadership has positively influenced engagement with ethical initiatives (87%, n=194/223 overall; 90.2%, n=92/102 men; 84.3%, n=102/121 women) and diversity and inclusion (89.7%, n=200/223 overall; 92.2%, n=94/102 men; 87.6%, n=106/121 women).

Women leaders cited multiple examples of prioritising their constituents’ health over financial profits by directly purchasing stock to ensure commodity availability, conducting additional community outreach in hard-to-reach areas and providing services directly, particularly for marginalised groups.

I want my integrity, and I want my space, and I want to do things correctly because at the end of the day, I want to see the impact of what I have done. (IDI 12, Zambia)

These approaches led to increased representation of marginalised groups in decision-making, resulting in improved programme inclusivity.

This [success] was driven by women. I am not sure how men would have done it, but I owe the successes in the HIV response to most of the women leaders … women just tend to be on the move, and they make a difference. While men are still trying to conceptualize what is this all about, what is in it for us, women would have already gone 10 miles away. (WL27_Eswatini_R)

Women leaders are not necessarily more ethical than men, but likely face additional scrutiny, needing to prioritise accountability and transparency to retain leadership roles.

Even though women often have to overcome more hurdles to reach leadership positions—where a man might need to cross five steps, a woman may need to cross ten. Having navigated these challenges, women leaders develop a strong sense of accountability and deliver with a high level of commitment and role modeling. (C3, P9, WL_Ethiopia_R&I)

### Creating a supportive environment for women leaders

Many women leaders actively seek to strengthen organisational capacity and structures to advance gender equality within their organisations and create a supportive environment for women leaders to thrive. These included activities to strengthen broader institutional structures and systems, such as advocating for equal pay, implementing sexual harassment policies, establishing flexible working policies, establishing family leave/maternity leave policies, establishing nursing rooms for working mothers, conducting training and capacity building activities and career and mentoring opportunities for women within the organisation.

Respondents strongly agreed/agreed that women’s leadership has positively influenced their organisational culture and climate (84.8%, n=190/224 overall; 86.4%, n=89/103 men; 83.5% n=101/121 women), a diverse and inclusive workforce and policies (81.7%, n=156/191 overall; 84.6%, n=77/91 men; 79%, n=79/100 women), training and development (84.7%, n=166/196 overall; 82.8%, n=77/93 men; 86.4%, n=89/103 women), career and mentoring opportunities for women (84.2%, n=165/196 overall; 87.1%, n=81/93 men; 81.6%, n=84/103 women) as well as employee retention (64.3%, n=144/224 overall; 71.6%, n=73/102 men; 58.2%, n=71/122 women) within their organisation or field. Responses to the gender pay gap were more diverse, where a sizeable proportion of both men (20.2%, n=18/89) and women (21.0%, n=21/100) strongly disagree/disagree that women leaders have positively influenced the gender pay gap.

[…] we have been working on company culture that can be documented with a policy for maternity leave and what that looks like, and giving fathers the opportunity to work from home for a few weeks after their babies are born. I think those things are all very progressive and add the message that we are then trying to send to larger corporations around. Allowing mothers to go and take their child to the clinic without having to take leave. […] historically have not been in place. (WL18_South Africa_R)

As a result, these measures can lead to increased job satisfaction, higher retention rates and improved productivity, ultimately ensuring that valuable talent is retained within the organisation.

### Intentional integration of gender within programmes and services

Integrating gender within programmes and services focuses on addressing gender inequities at the programme and service level. Gender integration activities championed and implemented by women leaders in our sample included ensuring women’s representation as beneficiaries, engaging women in the community as implementers and developing male engagement strategies.

The other way we have done so much is to be able to use the kind of mothers who had a positive impact to serve as peer educators to other mothers. […]. So, what we have done is when we work with your child and the child has a positive impact, as a mother, we involve you as part of the team. We have been involving them [mothers] to talk, to share their story, to be peer supporters. Some of them are the ones who are leading the mother-to-mother support group. (WL02_Cameroon_R)

According to our respondents, these initiatives contributed to more inclusive health services and programmes for children, adolescent girls and marginalised groups, including persons with disabilities and the empowerment of women within the community. In the example from Nigeria below, empowering women through their involvement in programme implementation directly helped to increase vaccination coverage within the community.

So, we ensure that the women groups, the adolescents, they are factored into our programmes…that there can be that gender representation in what we are doing. Because, understanding that when you leave the women behind, when you leave the girls behind, you can only grow a little. (WL05_Nigeria_I)

[…] there was even a project we conducted some time ago, I think it was in 2018, 2017, when we got to the community, … their vaccination was very low. Very low. When we got it from the state and data, we thought about the strategy to use, we said, okay, let’s use women. We empowered the women. The women became champions and got it done for us. By the end of the project, within six, not even at the end, within six months, it was increased. There was an evident 8% increase in two communities. (WL08_Nigeria_IR)

### Advocating for women’s representation within leadership roles

Within our sample, some women leaders actively promoted the inclusion of other women leaders in male-dominated leadership spaces. When asked about organisational outcomes, 84.4% (n=189/224) of respondents strongly agreed/agreed that women leaders have positively influenced opportunities for other women at their organisation, and men (84.5%, n=87/103) and women (84.3%, n=102/121) have equally positive views.

It really starts with identifying woman-leaders and the woman’s organizations, especially in the field that I work. Which is predominantly led by men. You know the medical field areas around public health, reproductive [health…]. The field is dominated by men, and most leaders in the field are men. So, we need to start by really prioritizing and looking for women. Because if we expect women will come to us, that has not been the reality. […] To look for woman-led organizations or woman leaders and to meet them where they are. (WL20_Ethiopia)

Increased representation of women in leadership generates a feedback loop where the presence of women in decision-making roles inspires and supports the rise of more women to leadership positions. This compounding effect amplifies the benefits and positive changes they bring to health, wellness and organisational effectiveness, creating a virtuous cycle of empowerment and progress.

Our analysis revealed no significant differences in perceptions between men and women regarding the impact of women leaders (table 2). A one-way ANOVA was performed to assess whether participants from different organisation types and African regions perceived the women leaders’ impact differently. Mean scores for organisational, health policy and gender outcomes were generally high across all participant groups. By gender, women reported mean scores of 4.03 (SD=0.60) for organisational outcomes, 3.99 (SD=0.59) for health policy outcomes and 4.03 (SD=0.66) for gender outcomes, while men reported 4.02 (SD=0.68), 3.99 (SD=0.73) and 4.14 (SD=0.68), respectively; these differences were not statistically significant. By primary affiliation, respondents from NGOs had the highest organisational outcome scores (mean=4.11, SD=0.62) compared with government (3.91, SD=0.62) and other organisations (4.01, SD=0.64), with this difference reaching statistical significance (F=3.33, p=0.037). For gender outcomes, NGO respondents scored 4.15 (SD=0.65) vs 3.92 (SD=0.71) for government and 4.09 (SD=0.64) for others, showing a trend towards higher perceived impact (F=2.83, p=0.060). Across African regions, mean organisational scores ranged from 3.98 (Southern Africa) to 4.07 (East Africa), health policy outcomes from 3.87 (Central Africa) to 4.04 (East Africa) and gender outcomes from 3.97 (Southern Africa) to 4.11 (East and West Africa), with no statistically significant differences. These results indicate that organisational affiliation, rather than gender or region, may be more strongly associated with perceptions of women leaders’ impact.

**Table 2:** Mean differences in organisational, overall and gender-related and health-related outcome scores stratified by gender, primary affiliation and African region

Background characteristics			Organisational outcomes				Health policy and programme outcomes				Gender outcomes		
		N	Mean	t/F	P value	N	Mean	t/F	P value	N	Mean	t/F	P value
Gender of respondent	Woman	230	4.03 (SD=0.6)	0.08	0.9400	194	3.99 (SD=0.59)	0.07	0.9425	155	4.03 (SD=0.66)	−1.38	0.1695
	Man	169	4.02 (SD=0.68)			149	3.99 (SD=0.73)			134	4.14 (SD=0.68)		
Primarily affiliated organisation type	Government	103	3.91 (SD=0.62)	3.33	0.0366*	81	3.96 (SD=0.65)	0.12	0.888042	69	3.92 (SD=0.71)	2.83	0.060459
	Non-governmental organisation	181	4.11 (SD=0.62)			158	4.00 (SD=0.7)			133	4.15 (SD=0.65)		
	Others	122	4.01 (SD=0.64)			110	4.00 (SD=0.59)			92	4.09 (SD=0.64)		
African region	Central Africa	130	4.03 (SD=0.65)	0.30	0.825	110	3.87 (SD=0.79)	2.32	0.075	94	4.08 (SD=0.71)	0.42	0.742
	East Africa	116	4.07 (SD=0.6)			101	4.04 (SD=0.57)			84	4.11 (SD=0.66)		
	Southern Africa	54	3.98 (SD=0.56)			46	4.04 (SD=0.5)			34	3.97 (SD=0.51)		
	West Africa	99	4.02 (SD=0.68)			86	4.09 (SD=0.55)			77	4.11 (SD=0.67)		

* Denotes <0.05 statistical significance.

## Discussion

Our results show that women leaders prioritise women-centric and neglected health issues, such as reproductive health, maternal health and gender-based violence. Without their leadership, these critical areas may not receive the necessary attention and resources, leading to poorer health outcomes for women and adolescent girls. This neglect can result in increased rates of maternal mortality, untreated reproductive health issues, increased unmet need for family planning, and a lack of support for adolescent girls navigating puberty and sexual health.^15 16^ Our results also show that women leaders have unique access to communities and can build trust with marginalised groups, ensuring that health services reach those who need them most. Without their involvement, these populations may remain underserved, leading to higher rates of preventable diseases and failure to achieve public health goals.

Without women leaders, we risk perpetuating persistent gaps in under-representation. Women leaders serve as role models and mentors, inspiring and supporting the next generation of women leaders and women with access to mentorship and peer networks are more likely to thrive and overcome challenges.^17^,^19^ Their absence means fewer women will be encouraged to pursue leadership roles, perpetuating a cycle of under-representation that can lead to policies and programmes that do not fully consider the needs and perspectives of women and marginalised groups.^20^,^22^ Furthermore, without women leaders’ influence, organisations may fail to create environments that are conducive to the success of women at all levels, resulting in the loss of valuable talent.^23^ This loss affects individuals and deprives organisations and communities of the unique contributions that women leaders bring.

Respondents affiliated with NGOs reported higher mean scores for organisational and gender outcomes compared with those from government and other organisations. This finding suggests that organisational culture and structure may play a more significant role in shaping perceptions of leadership impact than gender alone. Babalola *et al* noted that women in leadership positions in African Science, Technology, Engineering and Mathematics fields face challenges such as gender bias and a lack of supportive structures, which can hinder their leadership effectiveness.^24^

Women leaders are conducting their leadership differently, using novel approaches and strategies to influence health policy and programme change within their countries. At the same time, they influence health outcomes and impacts through many of the same mechanisms that a leader would use regardless of gender, such as through engagement in policy-making processes, engaging with decision-makers, fostering collaborations with international stakeholders, using evidence to inform decision-making, capacity building and role modelling. However, considering the unique contributions of women leaders to RMNCAH-N and immunisation efforts, the outcomes and impacts documented in this study would likely not have been achievable without them.

We mapped recommendations to each of the impact pathways shown in figure 1 to highlight ways in which women leaders’ impact can be amplified and enhanced towards continued improvement of health outcomes globally.

**Figure 2:**
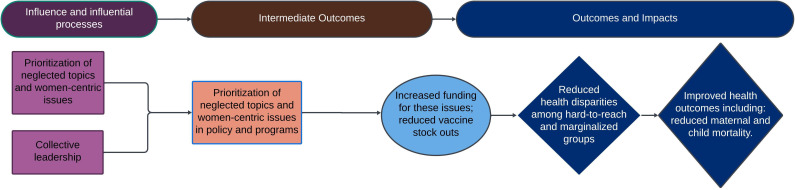
Impact pathway 1—prioritisation of women-centric and neglected issues.

Recommendation 1: women leaders should be supported to fundraise for the issues they prioritise, such as women-centric and neglected issues within policies and programmes. Women leaders are shown to effectively mobilise resources for issues that affect women and girls in their organisations and fields, which can lead to reduced health disparities among hard-to-reach and marginalised groups and improve health outcomes, including reduced maternal and child mortality (figure 2).

**Figure 3:**
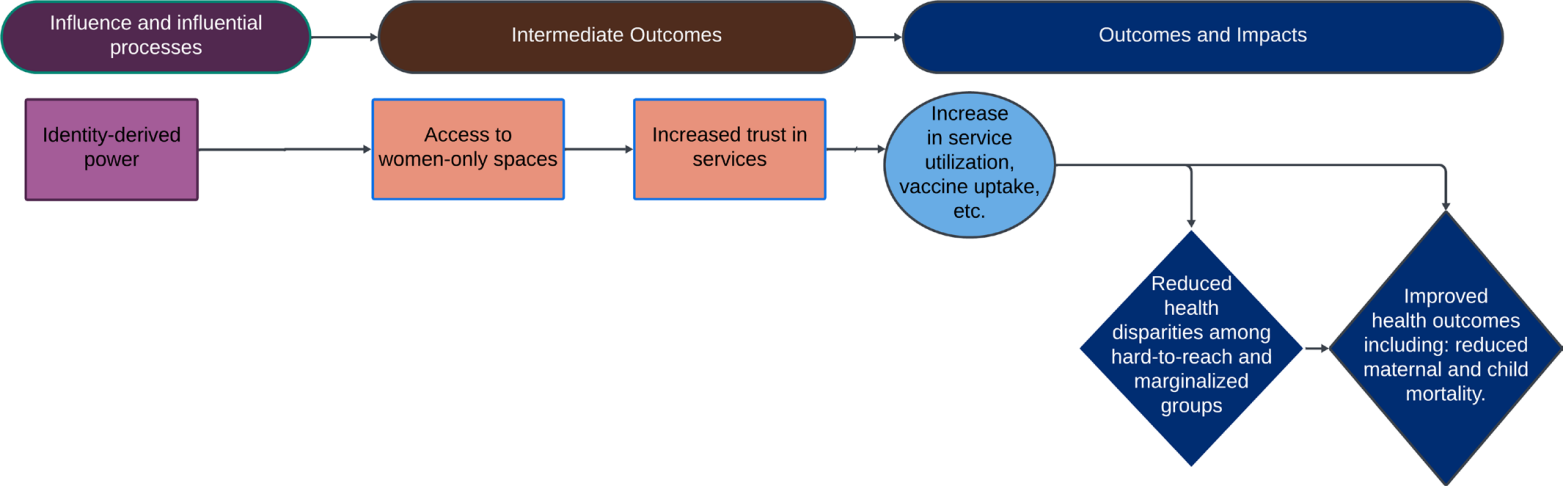
Impact pathway 2—identify-derived power.

Recommendation 2: women leaders should be supported to engage with communities. Their experiences as women, mothers and caregivers often enable them to access spaces within the community that men may not be able to access. This can increase communities’ trust in services, leading to an increase in service utilisation and intervention uptake (figure 3).

**Figure 4:**
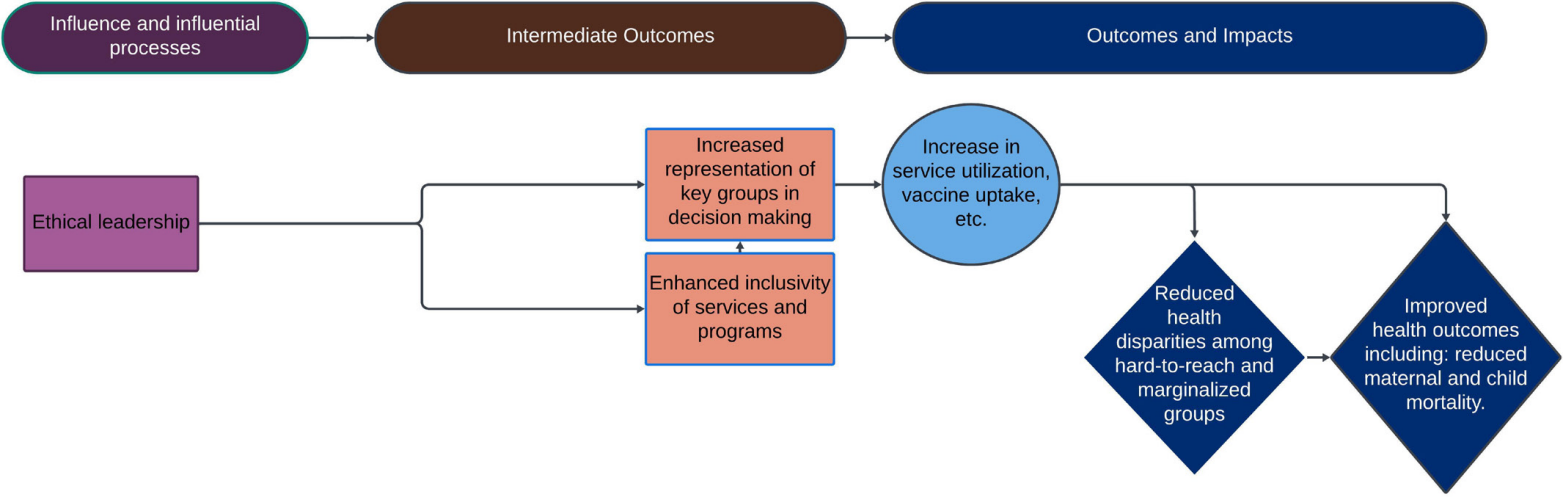
Impact pathway 3—ethical leadership.

Recommendation 3: women leverage principles of ethical leadership, leading to increased inclusivity of services and increased representation of key groups in decision-making. This leads to an increased use of services (figure 4). By embedding principles of transparency, accountability and inclusive decision-making in governance structures, we can foster more inclusive and representative service environments and ensure that all leaders, regardless of gender, equally engage in ethical leadership.

**Figure 5:**
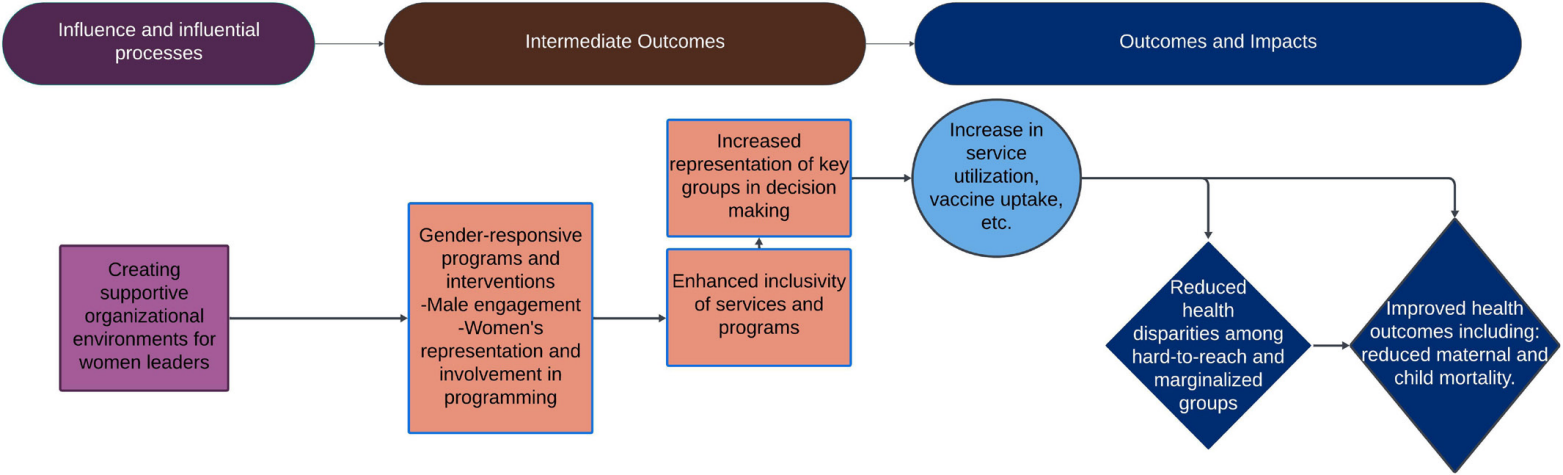
Impact pathway 4—gender integration.

Recommendation 4: gender integration activities implemented by women leaders, such as ensuring women’s representation as beneficiaries, engaging women in the community as implementors and developing male engagement strategies, should be championed. These initiatives can contribute to more inclusive health services and programmes for children, adolescent girls and marginalised groups, as well as the empowerment of women and girls within the community (figure 5).

**Figure 6:**
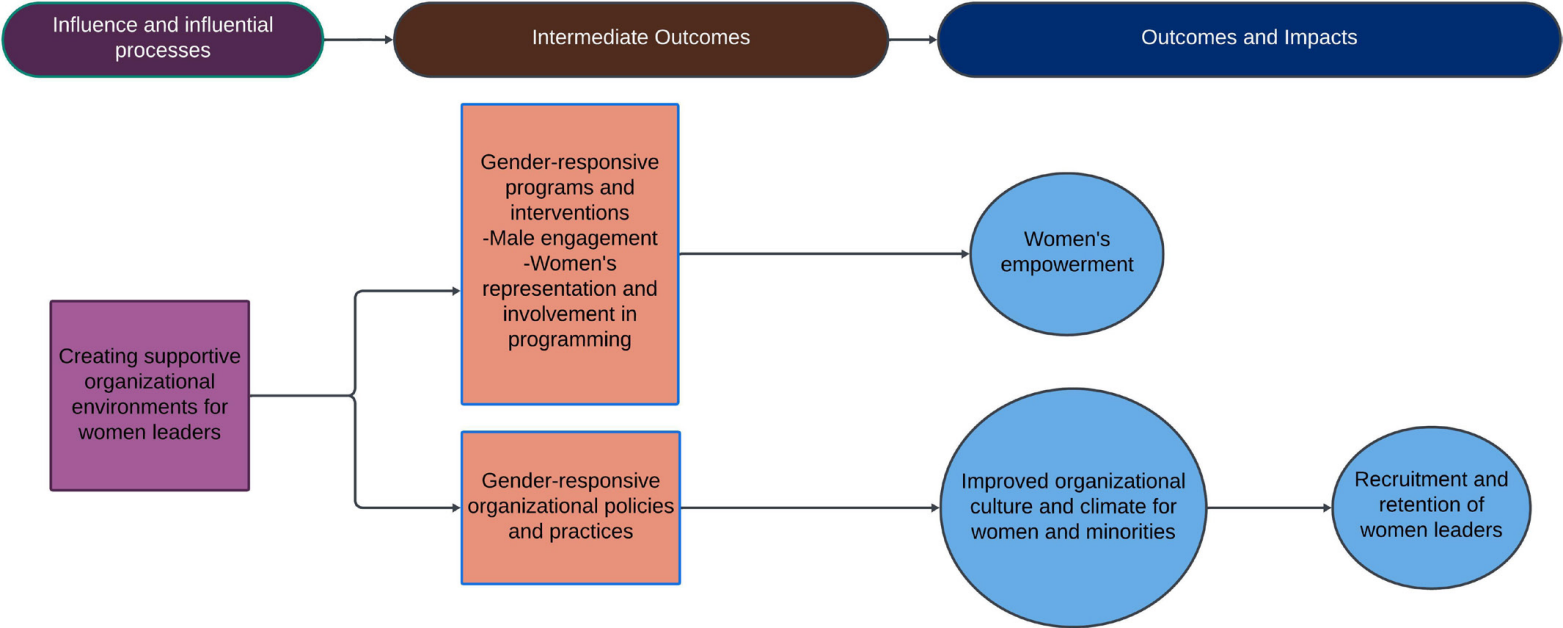
Impact pathway 5—creating supportive environments.

Recommendation 5: women leaders create supportive environments for other women, generating gender-responsive policies and programmes that improve organisational culture and support the ongoing recruitment, retention and growth of women leaders (figure 6). Women leaders should be supported in implementing initiatives and policies to create more enabling environments for women, ensuring that valuable talent is retained within the organisation.

Women leaders’ networks, often structured and sustained differently from men’s, continue to pose challenges in fully maximising their leadership impact. Women are less likely than men to have personal relationships with high-status coworkers, and in fields where women are under-represented, like immunisation, women experience worse networking outcomes.^25 26^ But our findings showed that women leaders use the power of the collective to overcome male-dominated decision-making spaces; they leverage their combined leadership power to promote agendas that might face resistance or ambivalence. Investing in women leaders’ networks through regional forums, convenings, curated communities of practice and training in advocacy and multisectoral collaboration can support robust networks of women leaders who can collectively drive impactful change, including within their organisations.

In parallel, organisations should actively cultivate male allyship as a core component of advancing equity. When men are engaged not as bystanders but as co-advocates, they can help shift organisational culture, challenge discriminatory practices and reinforce accountability.^27 28^ Organisational leadership should embed allyship into performance metrics, mentorship programmes and team dynamics—ensuring that gender equity is not a siloed responsibility, but a shared commitment across all levels.

### Limitations

This study was subject to limitations. Despite conducting comprehensive online stakeholder mapping, we were only able to reach out to people via email, which means that leaders without an online presence were missed. Additionally, the sample size was lower than expected, which was partly due to the length of the survey tool. The survey length led to a drop-off in respondents, as many who opened the survey did not complete it. Not all respondents answered all questions within the survey, which led to different sample sizes for each question. Because analyses were conducted per question using variable denominators, differences in item-level response rates may have influenced the precision of estimates and introduced potential non-response bias if missingness was not completely random. While this analytic approach was appropriate to retain available data, these limitations should be considered when interpreting the findings. Nevertheless, the generally high response rates across most items suggest that any resulting bias is unlikely to have materially affected the overall conclusions. In addition, the survey documented perceptions of impact rather than actual impact, which means we cannot report on actual impact.

It was challenging to recruit participants for the KIIs, likely because they had already completed the online survey, resulting in a smaller sample size than expected. Most women in this field are accustomed to discussing barriers to leadership, requiring interviewers to spend time priming and redirecting respondents away from barriers during the interviews. Women are also less likely to document the impact of their work at an individual level due to a combination of social, organisational and methodological factors, which may have made it difficult for them to fully express the impact of their leadership.

Despite these limitations, this study remains critically important to the field. By combining an online survey with KIIs, the study used a multifaceted approach to gathering insights, which helps provide a more nuanced understanding of the impact of women’s leadership on health.

## Conclusion

The findings from this study underscore the critical role of women leaders in advancing health policy and gender equity across SSA. Women leaders have demonstrated their ability to prioritise women-centric and neglected health issues, such as reproductive and maternal health, and gender-based violence, ensuring these areas receive the necessary attention and resources. Their unique access to communities and ability to build trust with marginalised groups have been instrumental in reaching hard-to-reach populations, thereby improving health outcomes. Investing in women leaders is essential to minimise health disparities, support and retain valuable talent and create enabling environments that foster the success of women in leadership roles. This investment will benefit women and adolescent girls and contribute to achieving broader public health goals and sustainable development. Further research is needed to explore the underlying mechanisms driving the reported perceptions and to develop strategies that promote inclusive and effective leadership across diverse contexts.

## Supplementary material

10.1136/bmjgh-2025-021163 online supplemental file 1

## Data Availability

Data are available on reasonable request.
