# Supplementary material for: Exploring the perceived impact and influence of women leaders across sub-Saharan Africa on health policy and gender equity
Source: BMJ Glob Health. 2025 Dec 19;10(12):e021163. doi: 10.1136/bmjgh-2025-021163 (PMC12716510; doi:10.1136/bmjgh-2025-021163)
Supplement: online supplemental file 1 [file bmjgh-10-12-s001.docx]

**Impact of Women's Leadership in RMNCAH-N and Immunization**

Johns Hopkins Bloomberg School of Public Health is working with the Global Financing Facility to generate evidence for investing in women’s leadership in global health and document the impact of this investment on reproductive, maternal, newborn, child, and adolescent health and nutrition (RMNCAH-N) and immunization outcomes. Participation in this study allows the study team to get a better understanding of your experience as a woman leader and/or with women leaders. You do not have to agree to be in this study, and you may change your mind at any time without any penalty or loss of benefit. Please review the details outlined in the rest of this consent document before deciding.

If you consent to join the study, you will be prompted to complete a survey on your experiences as a woman leader and/or with women leaders. The survey can be completed online individually, or on Zoom or on the phone with a member of the research team. The survey has two parts, and each part is anticipated to take between 20 and 30 minutes of your time, or up to 60 minutes in total. You may pause the survey after each part and resume at your convenience, within the indicated deadline. You may skip any question(s) in the survey that you do not wish to answer.

There is no direct benefit to you from being in this study, however the potential societal benefits are an investment in women’s leadership in RMNCAH-N and immunization, leading to more programs and resources for women everywhere. There is no cost for participating in the study, nor is there any payment or compensation for your participation. However, participants who complete both  parts of the survey will be able to enter a drawing for sponsorship for a professional development opportunity, preferably related to leadership (up to $800). By completing the survey, participants will be prompted to enter the drawing by entering their email on a webpage that is not linked with the survey responses. Efforts will be made to minimize privacy and confidentiality risks at different stages of data collection and data management. At the end of the survey, participants will be invited to voluntarily submit their contact information, in a separate unlinked form, in order to be contacted for a key informant interview.

*How we use and manage your personal data:*  All responses will be kept confidential and data will be de-identified. Names and organizational data will not be included in any analysis associated with this study. The data will be used for general analysis, policy formation and planning for your community. Every effort will be made to ensure confidentiality of the information you provide. However, there is a minimal potential for breach of confidentiality. There are no physical risks to this project. You do not need to answer every question; you may stop the survey or leave the project at any time. We ask that you complete the survey from a private location as you will be asked potentially sensitive questions. In the event that you complete the survey with a member of our research team, the researcher will also conduct the survey from a private location. There is a risk that someone outside the study will see your information. We will do our best to keep your information safe by keeping all data on password protected computers. All this material will only be accessible to members of this team. Participation, or not, will not affect your employment.

If you have any questions about this study please contact the Principal Investigator, Dr. Anna Kalbarczyk (akalbarc@jhu.edu). The JHU Bloomberg School of Public Health Institutional Review Board can be reached at: JHSPH.irboffice@jhu.edu .

Thank you for your interest in this survey. Once complete, we would like to invite you to enter a drawing to **win a scholarship or sponsorship to a professional development opportunity of your choice** **(ideally related to leadership), up to $800 USD**! You will receive a prompt to enter the drawing at the end of the survey. In order to be eligible for the drawing, you must complete Section One of the survey and add at least one member to the Social Network Analysis component of the survey.

*Please note:this is entirely voluntary, and your drawing will in no way be linked to your survey responses.*

Do you consent to participate in the study?

- Yes
- No

Skip To: End of Survey If Do you consent to participate in the study? = No

Are you 18 years or older?

- Yes
- No

Skip To: End of Survey If Are you 18 years or older? = No

Q1 Please select which country you are a national of or where your primary citizenship is from:

- Angola
- Burundi
- Benin
- Burkina Faso
- Botswana
- Central African Republic
- Côte d’Ivoire
- Cameroon
- Congo, Dem. Rep.
- Congo, Rep.
- Comoros
- Cabo Verde
- Eritrea
- Ethiopia
- Gabon
- Ghana
- Guinea
- The Gambia
- Guinea-Bissau
- Equatorial Guinea
- Kenya
- Liberia
- Lesotho
- Madagascar
- Mali
- Mozambique
- Mauritania
- Mauritius
- Malawi
- Namibia
- Niger
- Nigeria
- Rwanda
- Sudan
- Senegal
- Sierra Leone
- Somalia
- South Sudan
- São Tomé and Príncipe
- Eswatini
- Seychelles
- Chad
- Togo
- Tanzania
- Uganda
- South Africa
- Zambia
- Zimbabwe
- None of the above

Skip To: End of Survey If Please select which country you are a national of or where your primary citizenship is from: = None of the above

Q2 Do you or have you ever lived and worked in sub-Saharan Africa at least 80% of the time?

- Yes
- No

Skip To: End of Survey If Do you or have you ever lived and worked in sub-Saharan Africa at least 80% of the time? = No

Q3 If yes, in which count(ries) do you or did you live and work in?

________________________________________________________________

Q4 Do you currently work in or have recently worked in reproductive, maternal, newborn, child, or adolescent health, nutrition (RMNCAH-N) or Immunization? Select all that apply:

- Reproductive, maternal, newborn, child, or adolescent health, nutrition
- Maternal and newborn health, including antenatal and postnatal care, lactation, premature and low-birth weight infants, and stillbirths
- Child health, including pediatrics and early childhood development
- Reproductive health, including family planning, cervical cancer, infertility, and STIs
- Adolescent Sexual and Reproductive Health and Rights (ASRHR)
- Nutrition
- Immunization
- Pharmaceutics, including vaccine distribution
- Epidemiology
- Vaccine promotion / outreach
- Medicine and nursing, including vaccine administration
- Vaccine Supply Chain
- Other RMNCAH-N or immunization work. Please indicate below: ____________
- No

Skip To: End of Survey If Do you currently work in or have recently worked in reproductive, maternal, newborn, child, or ad... = No

Q5 In your current organizational or institutional role, do you have influence over one or more of the following: - How decisions are made? - Which priorities are identified? - How funding is distributed? - The strategic direction?

- Yes
- Don't Know
- No

Skip To: End of Survey If In your current organizational or institutional role, do you have influence over one or more of t... = No

Skip To: End of Survey If In your current organizational or institutional role, do you have influence over one or more of t... = Don't Know

Start of Block: Demographics

Q6 What is your race?

- Black
- Mixed race
- Indian
- Asian
- White
- Other race not listed. Please indicate below: _________________________________

Q7 What is your gender?

- Man
- Woman
- Other gender not listed. Please indicate below: _______________________________

Q8 How old are you? _________________________________________________________

Q9 What type of organization are you primarily affiliated with?

- Government
- University
- Non-governmental organization
- Faith-based organization
- Private sector
- Multilateral organization
- Organization type not listed above. Please indicate below: _______________________

Q10 What level would you characterize your current role?

- Community / Facility
- District
- Provincial
- National
- Not applicable

Q11 What is your primary role within your organization?

- Economist
- Epidemiologist
- Researcher
- Minister of Health
- Chief of Mission
- Chief of Party
- Representative
- Advisor / Advisory Group Member
- Head of Department
- Head of Office
- Country Director
- Regional Director
- Subregional Director
- Deputy Regional Director
- Program Director
- Health Facility Director
- District Manager
- EPI Manager
- Supply Chain Manager
- Program Manager
- National Coordinator
- Project Coordinator
- Finance Officer
- Program Officer
- Senior Specialist / Associate / Officer
- Other role. Please indicate below: __________________________________________

Start of Block: Section 1: Perceptions of women leaders’ impact across SSA

Q14 This section explores the type(s) of impact that women leaders have had in RMNCAHN and immunization.

Q13 **Organizational outcomes:**  Women leaders can be represented at multiple levels within an organization. Consider women leaders’ overall participation within the organization when answering this question. For each of the items below please indicate the extent to which you agree or disagree with the statement, “Women’s leadership in RMNCAHN and/or Immunization has positively influenced....”.

|  | Strongly Disagree | Disagree | Neither Disagree or Agree | Agree | Strongly Agree | N/A |
| --- | --- | --- | --- | --- | --- | --- |
| My organization’s **financial performance** (I.e is more profitable, receives more donor funding, is more cost-effective/efficient). |  |  |  |  |  |  |
| My organization’s **competitiveness** (I.e winning bids, getting grants, making partnerships, etc). |  |  |  |  |  |  |

| My organization’s **employee retention**. |  |  |  |  |  |  |
| --- | --- | --- | --- | --- | --- | --- |
| My organization’s **employee performance** (I.e. forging more and/or stronger partnerships, exhibiting creative thinking, etc.). |  |  |  |  |  |  |
| My organization’s **engagement with ethical initiatives** (I.e. contributes to order and harmony in society, community wellbeing, guiding younger generations, living in harmony with nature, corporate social responsibility (CSR), etc.). |  |  |  |  |  |  |
| My organization’s **organizational culture/climate** (I.e. employee moral/job satisfaction, motivation, communication, and teamwork, ensured safety of health workers, training of vaccinators and social mobilizers, prompt payment of healthcare worker). |  |  |  |  |  |  |
| My organization’s **diversity and inclusion** (I.e. there are equal opportunities and a sense of belonging for people of all race, ethnicities, genders, and ages). |  |  |  |  |  |  |
| My organization’s **innovation** (I.e. introduces new ideas, services, products, processes, or methods). |  |  |  |  |  |  |
| **Opportunities for other women** at my organization (I.e for promotion, raises, career/ mentoring opportunities, leadership opportunities, professional development opportunities, and/or taking on more responsibilities). |  |  |  |  |  |  |

Q15 Because of women’s leadership, my organization has experienced other benefits not listed here. Please explain.

________________________________________________________________

________________________________________________________________

________________________________________________________________

________________________________________________________________

________________________________________________________________

Q16 **Extent of impact:**  In your opinion, to what extent have women leaders had an impact on each of the following areas...

|  | Not at all | Very little | | Somewhat | | | To a great extent | | N/A | |
| --- | --- | --- | --- | --- | --- | --- | --- | --- | --- | --- |
| **Financial performance** in your organization (I.e is more profitable, receives more donor funding, is more cost-effective/efficient). |  |  | |  | | |  | |  | |
| **Competitiveness** in your organization (I.e winning bids, getting grants, making partnerships, etc). |  |  | |  | | |  | |  | |
| **Employee retention** in your organization. |  |  | |  | | |  | |  | |
| **Employee performance** in your organization (I.e. forging more and/or stronger partnerships, exhibiting creative thinking, etc.). |  |  | |  | | |  | |  | |
| **Engagement with ethical initiatives** in your organization (I.e. contributes to order and harmony in society, community wellbeing, guiding younger generations, living in harmony with nature, corporate social responsibility (CSR), etc.) |  |  | |  | | |  | |  | |
| **Organizational culture/climate** (I.e. employee moral/job satisfaction, motivation, communication, and teamwork, ensured safety of health workers, training of vaccinators and social mobilizers, prompt payment of healthcare worker). |  |  | |  | | |  | |  | |
| **Diversity and inclusion** in your organization (I.e. there are equal opportunities and a sense of belonging for people of all race, ethnicities, genders, and ages). |  |  | |  | | |  | |  | |
| For this item, check the option "**Very little**" |  | |  | |  |  | |  | |  |
| **Innovation** in your organization (I.e. introduces new ideas, services, products, processes, or methods). |  | |  | |  |  | |  | |  |
| **Opportunities for other women** in your organization (I.e for promotion, raises, career/ mentoring opportunities, leadership opportunities, professional development opportunities, and/or taking on more responsibilities). |  | |  | |  |  | |  | |  |

Q17 **Outcomes:**  For each of the items below, please indicate the extent to which you agree with the statement, “Women’s leadership in RMNCAHN and/or Immunization has positively influenced:”

|  | Strongly Disagree | Disagree | Neither Disagree or Agree | Agree | Strongly Agree | N/A |
| --- | --- | --- | --- | --- | --- | --- |
| **Health outcomes** for the populations with which I work. (i.e. immunization coverage, ANC uptake, adolescent fertility rate, reduction of malnutrition, access to modern contraception) |  |  |  |  |  |  |

| **Policy decisions** for the populations with which I work. (i.e. legalizing new contraception or abortion, improving women’s rights and access to the legal system, adding a new immunization to EPI, other policies related to immunization or RMNCAHN) |  |  |  |  |  |  |
| --- | --- | --- | --- | --- | --- | --- |
| **Financial security or opportunity** for the community and/or populations with which I work. (I.e. economic empowerment, reduction of costs, or access to social protection mechanisms to alleviate the costs) |  |  |  |  |  |  |
| Facilitating **access to decision-making bodies/ forums for the health sector** for the community and/or populations with which I work. |  |  |  |  |  |  |
| **Organizational culture/climate** within my organization (I.e. Employee satisfaction, retention, prioritization of safety of health workers) |  |  |  |  |  |  |
| **Training and development** within my organization and/or field (I.e. training of vaccinators and social mobilizers, etc.) |  |  |  |  |  |  |
| **Career/mentoring opportunities** for women within my organization and/or field |  |  |  |  |  |  |
| **The gender pay gap** within my organization and/or field (I.e. reduced the gap between women and men) |  |  |  |  |  |  |
| **Diverse and inclusive workforce and policies** within my organization and/or field. |  |  |  |  |  |  |

Q18 Because of women’s leadership, the community or populations with which I work, my organization and/or field have experienced other benefits not listed here. Please explain.

________________________________________________________________

________________________________________________________________

________________________________________________________________

________________________________________________________________

________________________________________________________________

Q19 **Extent of impact:**  In your opinion, what extent have women leaders had an impact on each of the following areas …

|  | Not at all | Very little | Somewhat | To a great extent | N/A |
| --- | --- | --- | --- | --- | --- |
| **Health outcomes** for the populations with which I work. (i.e. immunization coverage, ANC uptake, adolescent fertility rate, reduction of malnutrition, access to modern contraception) |  |  |  |  |  |
| **Policy decisions** for the populations with which I work. (i.e. legalizing new contraception or abortion, improving women’s rights and access to the legal system, adding a new immunization to EPI, other policies related to immunization or RMNCAHN) |  |  |  |  |  |
| **Financial security or opportunity** for the community and/or populations with which I work. (I.e. economic empowerment, reduction of costs, or access to social protection mechanisms to alleviate the costs) |  |  |  |  |  |

| Facilitating **access to decision-making bodies/forums** for the community and/or populations with which I work. |  |  |  |  |  |
| --- | --- | --- | --- | --- | --- |
| **Organizational culture/climate** within my organization (I.e. Employee satisfaction, retention, prioritization of safety of health workers) |  |  |  |  |  |
| **Training and development** within my organization and/or field (I.e. training of vaccinators and social mobilizers, etc.) |  |  |  |  |  |
| **Career/mentoring opportunities for women** within my organization and/or field |  |  |  |  |  |
| **The gender pay gap** within my organization and/or field (I.e. reduced the gap between women and men). |  |  |  |  |  |
| **Diverse and inclusive workforce and policies** within my organization and/or field. |  |  |  |  |  |

Q20 **Women’s Health and Gender Outcomes:**  For each of the items below, please indicate the extent to which you agree with the statement, “Women’s leadership in RMNCAHN and/or Immunization has positively influenced:”

|  | Strongly Disagree | Disagree | Neither Agree or Disagree | Agree | Strongly Agree | N/A |
| --- | --- | --- | --- | --- | --- | --- |
| The **prioritization of key issues related to women and girls** in my area of work. |  |  |  |  |  |  |
| Increased **investment/ budget allocation in activities related to women and girls** within my organization and/or field. |  |  |  |  |  |  |
| Prioritization of **investments/ budget allocation on addressing gender barriers or reinforcing positive gender norms** within my organization and/or field. |  |  |  |  |  |  |
| **Development of gender policy/policies** within my organization and/or field. |  |  |  |  |  |  |
| **Integration of gender into relevant policies** within my organization and/or field. |  |  |  |  |  |  |
| Increased **partnerships** with other organizations to **understand gender-related issues in health and have experience in removing gender related barriers** within my organization and/or field. |  |  |  |  |  |  |
| Increased **establishment of women-centered institutions** within my field. |  |  |  |  |  |  |
| For this item, check the option "**Disagree**" |  |  |  |  |  |  |

| Improved women’s **involvement in the decision-making bodies, forums, governing boards** within my field. |  |  |  |  |  |  |
| --- | --- | --- | --- | --- | --- | --- |
| Reduction in **harmful gender norms and practices** (i.e. child marriage, Female Genital Cutting) for the communities or populations with which I work. |  |  |  |  |  |  |
| Reduced **gender-based violence** for the communities or populations with which I work. |  |  |  |  |  |  |
| Increased **male engagement** in health for the communities or populations with which I work. |  |  |  |  |  |  |
| Increase **autonomy and decision-making power** of women and girls for the communities or populations with which I work. |  |  |  |  |  |  |

Q21 Because of women’s leadership, the community or populations with which I work, my organization and/or field have experienced other benefits not listed here. Please explain.

________________________________________________________________

________________________________________________________________

________________________________________________________________

________________________________________________________________

________________________________________________________________

Q22 **Extent of impact:**   In your opinion, to what extent have women leaders had an impact on each of the following areas…

|  | Not at all | Very little | Somewhat | To a great extent | N/A |
| --- | --- | --- | --- | --- | --- |
| The **prioritization of key issues** related to women and girls in my area of work. |  |  |  |  |  |
| Increased **investment/ budget allocation in activities related to women and girls** within my organization and/or field. |  |  |  |  |  |
| Development of **gender policy/policies** within my organization and/or field. |  |  |  |  |  |
| **Integration of gender into relevant policies** within my organization and/or field. |  |  |  |  |  |
| Increased **partnerships with other organizations to understand gender-related issues in health and have experience in removing gender related barriers** within my organization and/or field. |  |  |  |  |  |
| Increased **establishment of women-centered institutions** within my field. |  |  |  |  |  |
| Improved **women’s involvement in decision-making bodies, forums, governing boards** within my field. |  |  |  |  |  |
| Reduction in **harmful gender norms and practices** (i.e. child marriage, Female Genital Cutting) for the communities or populations with which I work. |  |  |  |  |  |
| Reduced **gender-based violence** for the communities or populations with which I work. |  |  |  |  |  |

| Increased **male engagement** in health for the communities or populations with which I work. |  |  |  |  |  |
| --- | --- | --- | --- | --- | --- |
| Increase **autonomy and decision-making power** of women and girls for the communities or populations with which I work. |  |  |  |  |  |

Q23 To what extent do you agree with the statement, “I am able to have the type of impact that I would like **in my organization**”.

- Strongly disagree
- Disagree
- Agree
- Strongly Agree

Q24 What would enable you/ women leaders to have greater impact in your/ their organization (i.e. flexible work schedules, family-friendly policies, women’s representation, transparent pay structures, etc.)?

________________________________________________________________

________________________________________________________________

________________________________________________________________

________________________________________________________________

________________________________________________________________

Q25 To what extent do you agree with the statement “I am able to have the type of impact that I would like **in my field of work**”.

- Strongly disagree
- Disagree
- Agree
- Strongly agree

Q26 What would enable you/ women leaders to have greater impact in your/ their field of work (i.e. technical training/development, access to networks, women’s representation in decision-making bodies)?

________________________________________________________________

________________________________________________________________

________________________________________________________________

________________________________________________________________

________________________________________________________________

Start of Block: End of Section

Q28 This survey includes a social-network analysis. Would you like to proceed to the next section?

- Yes, please continue
- Not now, but I am willing to continue later *(Note: this will trigger an invite to the email reminder opt-in form)*
- No, I am done. (*Please note you will not be eligible for the sponsorship drawing without adding at least one member to to the Social Network Analysis.)*

Skip To: End of Survey If This survey includes a social-network analysis. Would you like to proceed to the next section? = No, I am done. (<em>Please note you will not be eligible for the sponsorship drawing without adding at least one member to to the Social Network Analysis.)</em>

Display this question:

If This survey includes a social-network analysis. Would you like to proceed to the next section? = Not now, but I am willing to continue later (Note: this will trigger an invite to the email reminder opt-in form)

Please provide your email by following this link in order to receive periodic reminders to complete this survey. You may pick up the survey where you left off, as long as you use the same internet browser and same computer that you started it with.

Start of Block: Social Network Analysis

Q29 **Social Network Analysis:**

In this section, we will explore who leaders interact with. We are particularly interested in relationships based on the exchange of advice or professional support, how strong those ties are, and what women leaders’ perceptions are of their impact, professional influences, and opportunities for leadership development.

We would like you to think of **at least five members of your network** who are important to you/you engage with in your career. While we request a minimum of five members, you can submit up to ten members of your network. Please proceed to the next page to begin the Social Network Analysis.

*Note: This section requests substantial demographic information including your name and contact information in addition to the names and contact information of individuals in your network. This information will be used to support respondent-driven sampling. All data will be de-identified in analysis and names and contact information will be kept separate.*

Q30 **Women Leaders’ Network Population:**

First, think of a member of your network who is important to you/you engage with in your career. They may provide information, support, or advice, or have influenced your career in some way. Please provide the following information:

|  | Network Member 1 |
| --- | --- |
| Name |  |
| Email (optional) |  |
| Country of residence |  |

Q29 What is this person's gender?

- Male
- Female
- Other gender not listed: __________________________________________________

Q31 What is their career stage?

- Student
- Trainee
- Early professional
- Mid professional
- Sr. Professional
- Retired

Q32 What type of organization are they currently affiliated with?

- Government
- University
- Non-governmental organization
- Faith-based organization
- Private sector
- Multilateral organization
- Organization type not listed above (fill in option)

Q33 What level would you characterize their current or past role?

- Community / Facility
- District
- Provincial
- National
- Not applicable

Q34 What field of work are they in?

- RMNCAH-N
- Immunization
- Both RMNCAH-N and Immunization
- Neither RMNCAH-N nor immunization

Q38 How frequently do you communicate with this person?

- About a few times a year
- About once a month
- About once a week or more

Q36 How well/how efficiently do you share resources (information, guidance, data, funding, contacts/networks) in this relationship?

- Not very well
- Somewhat /moderately well
- Very well

Q37 How much do you value this relationship?

- A little
- Moderately
- A lot

Q35 To what extent is this person able to make change or has made change in their organization and/or field?

- Minimally able to
- Somewhat able to
- Very able to

Q36 Would you like to add another member of your network? *Please remember that we would like you to provide information for at least five members of your network, but you may submit up to 10.*

- Yes
- No

Skip To: End of Block If Would you like to add another member of your network? Please remember that we would like you to p... = No

**[REPEAT FOR 10 NETWORK MEMBERS]**

Start of Block: SNA Part 2

Thank you for providing information about the members of your network who are important to you/you engage with in your career! Please continue below for our final set of questions:

Q39 How strong do you think your entire network is?

- Very strong
- Strong
- Weak
- Very Weak

Q40 What is the value of networks to you and the impact you can have in your organization and/or field?

________________________________________________________________

________________________________________________________________

________________________________________________________________

________________________________________________________________

________________________________________________________________

Q41 To what extent do you feel you are able to leverage your network to help meet your objectives, including:

|  | Very unable to | Unable to | Able to | Very able to | N/A |
| --- | --- | --- | --- | --- | --- |
| To advance your career |  |  |  |  |  |
| To improve your organization |  |  |  |  |  |
| To design/change/improve programs and policies |  |  |  |  |  |
| To affect health outcomes |  |  |  |  |  |
| To affect gender outcomes |  |  |  |  |  |
| Other: |  |  |  |  |  |

Q42 What do you need / want to strengthen your network?

________________________________________________________________

________________________________________________________________

________________________________________________________________

________________________________________________________________

________________________________________________________________

Start of Block: Incentive & KII Sign-Up

**Thank you!**

As a thank you for your time and energy spent completing this survey, we would now like to invite you to enter a drawing to win a scholarship or sponsorship to a professional development opportunity of your choice (ideally related to leadership), up to $800 USD. This is entirely voluntary, and your drawing will in no way be linked to your survey responses. You may enter the draw***here***.

Q43 **Would you be interested in being part of a key informant interview?**

The KII will discuss in more detail some of your responses to this survey.If you are interested in potentially being contacted to participate in an interview, please submit your name and contact information ***here***or directly email Katherine Banchoff, kbanchoff@jhu.edu.

End of Survey
